# Changes in systemic immune–inflammation index predict periprosthetic joint infection after hemiarthroplasty in elderly patients

**DOI:** 10.5194/jbji-11-387-2026

**Published:** 2026-07-03

**Authors:** Emre Bilgin, Onur Gultekin, Murat Kilic, Umit Burak Alparslan, Mehmet Berke Yusan, Ahmet Onur Akpolat

**Affiliations:** 1 Department of Orthopedics and Traumatology, University of Health Sciences, Fatih Sultan Mehmet Training and Research Hospital, Istanbul, Türkiye; 2 Department of Orthopedics and Traumatology, Civril State Hospital, Denizli, Türkiye; 3 Department of Orthopedics and Traumatology, University of Health Sciences, Haseki Training and Research Hospital Istanbul, Türkiye

## Abstract

**Background**: Periprosthetic joint infection (PJI) remains a devastating complication after hemiarthroplasty in elderly patients with femoral neck fractures. Early identification of high-risk patients is important. The systemic immune–inflammation index (SII), calculated as platelet × neutrophil / lymphocyte count, is a marker of systemic inflammation, although its role in this population remains unclear. **Methods**: This retrospective single-centre study included patients aged ≥65 years who underwent hemiarthroplasty between 2015 and 2025. Preoperative and postoperative day-5 SII values were calculated from routine blood tests. Δ SII was defined as the difference between postoperative and preoperative values. The primary outcome was PJI within 1 year after surgery. Logistic regression and receiver operating characteristic (ROC) analyses were performed. **Results**: A total of 976 patients were included, of whom 47 (4.8 %) developed PJI. Postoperative SII values were significantly higher in patients with PJI, whereas preoperative SII was not associated with infection risk. In multivariable analysis, postoperative SII remained independently associated with PJI (OR 1.195, 95 % CI 1.144–1.248, p<0.001 ). Δ SII was also independently associated with PJI (OR 1.124, 95 % CI 1.085–1.164, p<0.001 ). ROC analysis demonstrated good discriminatory performance for postoperative SII (AUC 0.882), with an optimal cut-off value of 2118. In contrast, Δ SII showed moderate discriminatory performance (AUC 0.764), with an optimal cut-off value of 656. **Conclusions**: Postoperative SII and Δ SII were independently associated with PJI after hemiarthroplasty. SII may aid early postoperative risk stratification and clinical decision-making when interpreted alongside clinical findings.

## 1 Introduction

Periprosthetic joint infection (PJI) is one of the most devastating complications following hip arthroplasty and is associated with prolonged hospitalization, repeated surgical interventions, impaired functional outcomes, and increased mortality (Sharoff et al., 2024; Roerink et al., 2024). The incidence of PJI following hip arthroplasty for traumatic fractures is higher than that observed after elective procedures (Natsuhara et al., 2019). Several factors may contribute to this increased risk, including advanced age, frailty, nutritional deficiencies, immune dysfunction, and the inflammatory response triggered by both trauma and surgery (Ojeda-Thies et al., 2024). In geriatric patients, age-related chronic low-grade inflammation (inflammaging) may influence the body's response to infection, highlighting the importance of early identification of high-risk individuals.

In recent years, systemic inflammatory and immune-based biomarkers derived from routine blood tests have gained attention as predictors of postoperative complications (Guo et al., 2025; Pang et al., 2025). The systemic immune–inflammation index (SII), calculated using platelet, neutrophil, and lymphocyte counts, has emerged as a marker of systemic inflammatory and immune status (Hu et al., 2014). Initially described in oncologic populations, SII has been shown to predict prognosis and complications in patients with malignancies, cardiovascular disease, and critical illness. Higher SII values are characterized by increased neutrophil and platelet counts together with lower lymphocyte counts, reflecting a stronger systemic inflammatory response that may be associated with an increased risk of infection (Gao et al., 2019).

Within orthopaedic surgery, evidence indicates that elevated systemic inflammatory indices are associated with adverse outcomes following elective joint arthroplasty (Kapadia et al., 2016). However, most studies focus on elective procedures and short-term outcomes, and data on the predictive value of SII for PJI remain limited (Kürüm et al., 2024). Evidence specifically addressing geriatric patients undergoing hip arthroplasty for traumatic fractures is lacking. While preoperative inflammatory markers have been evaluated (Liu et al., 2024; Alsabani et al., 2023), the clinical significance of postoperative indices obtained before discharge remains unclear. We therefore hypothesized that elevated postoperative SII levels would be associated with an increased risk of PJI in this patient population and may help identify patients at higher risk during the early postoperative period.

The aim of the present study was to investigate the association between preoperative and early postoperative SII values and the development of PJI within 1 year following hemiarthroplasty for traumatic hip fractures in geriatric patients.

## 2 Methods

This retrospective single-centre study was conducted after approval from the local institutional review board.

### 2.1 Study population

The study included patients aged 65 years and older who underwent hemiarthroplasty for femoral neck fractures between January 2015 and December 2025. Patients younger than 65 years were excluded ( n=23 ). Additionally, one patient with missing key variables required for multivariable analysis was excluded, resulting in a final cohort of 976 patients.

Exclusion criteria included active infection at the time of surgery, pathological fractures, polytrauma requiring prolonged intensive care, known hematologic or inflammatory diseases affecting blood cell counts, active malignancy under treatment, chronic immunosuppressive therapy, and incomplete laboratory or follow-up data.

### 2.2 Surgical technique

All patients underwent hemiarthroplasty using a standardized posterior approach as part of routine clinical practice at our institution.

### 2.3 Data collection

Demographic and clinical data were obtained from electronic medical records. Variables included age, sex, body mass index (BMI), American Society of Anesthesiologists (ASA) score, Charlson comorbidity index, diabetes mellitus, and length of hospital stay (Pollmann et al., 2020).

Laboratory parameters were derived from routine complete blood count measurements. Preoperative samples were obtained within 24 h prior to surgery, and postoperative samples were obtained on postoperative day 5 prior to discharge, in accordance with institutional protocols.

Postoperative clinical management and discharge decisions were not influenced by SII values as these markers were not routinely used in clinical decision-making during the study period.

### 2.4 Systemic immune–inflammation index

The systemic immune–inflammation index (SII) was calculated as platelet count × neutrophil count / lymphocyte count. Preoperative and postoperative day-5 SII values were calculated for each patient. The change in SII ( Δ SII) was defined as the difference between postoperative and preoperative values. Hematological parameters were obtained from complete blood count analyses performed using an automated hematology analyser (Mindray BC-6800, Mindray Bio-Medical Electronics Co., Shenzhen, China).

### 2.5 Perioperative antibiotic prophylaxis

All patients received perioperative antibiotic prophylaxis according to institutional protocols. A single intravenous dose of cefazolin was administered approximately 30 min prior to skin incision. No additional postoperative prophylaxis was routinely administered unless clinically indicated.

### 2.6 Outcome assessment

Follow-up data were obtained through outpatient visits and review of electronic medical records. Patients were categorized according to the presence (PJI group) or absence of PJI (non-PJI group) within 1 year after surgery.

Periprosthetic joint infection was diagnosed according to the 2018 International Consensus Meeting (ICM) criteria based on clinical findings, laboratory parameters, microbiological results, and radiological evaluation. Patients meeting these criteria during the 1-year follow-up were classified as having PJI (Parvizi et al., 2018; Bingham et al., 2018).

### 2.7 Statistical analysis

Continuous variables were assessed for normality and presented as mean ± standard deviation or median (interquartile range), as appropriate. Categorical variables were expressed as frequencies and percentages.

Baseline characteristics were compared between patients with and without PJI using the independent-sample t test or the Mann–Whitney U test for continuous variables and the chi-square test for categorical variables.

Univariable logistic regression analysis was performed to identify variables associated with PJI. Variables considered to be clinically relevant were subsequently included in multivariable logistic regression models to evaluate independent predictors of PJI.

Receiver operating characteristic (ROC) curve analysis was performed to assess the discriminatory ability of postoperative SII. The optimal cut-off value was determined using the Youden index, and sensitivity, specificity, positive predictive value, and negative predictive value were calculated.

Variables demonstrating internal inconsistency during data validation, including perioperative transfusion status, were excluded from the primary regression models. Statistical significance was defined as a p value < 0.05. All analyses were performed using IBM SPSS Statistics (IBM Corp., Armonk, NY, USA).

## 3 Results

A total of 1000 patients were initially identified in the database. After exclusion of 23 patients aged under 65 years and 1 additional patient with missing key variables required for multivariable analysis, 976 patients were included in the final analysis. Among these, 47 patients (4.8 %) developed periprosthetic joint infection within 1 year postoperatively.

Among the 47 patients who developed PJI, early postoperative infection was the most common subtype (59.6 %), followed by late acute (23.4 %) and chronic infection (17.0 %). *Staphylococcus aureus* and *Staphylococcus epidermidis* were the most frequently identified pathogens. The distribution of infection subtypes and causative microorganisms is presented in Table 1.

**Table 1:** Distribution of infection subtypes and causative microorganisms among patients with periprosthetic joint infection.

Infection subtype/microorganism	n (%)
Infection subtype	
Early postoperative infection	28 (59.6)
Late acute infection	11 (23.4)
Chronic infection	8 (17.0)
Causative microorganism	
*Staphylococcus aureus*	12 (25.5)
*Staphylococcus epidermidis*	9 (19.1)
Culture-negative infection	6 (12.8)
Polymicrobial infection	5 (10.6)
*Escherichia coli*	5 (10.6)
*Staphylococcus haemolyticus*	3 (6.4)
*Acinetobacter baumannii*	2 (4.3)
*Enterococcus faecium*	2 (4.3)
*Klebsiella oxytoca*	2 (4.3)
*Serratia marcescens*	1 (2.1)

**Figure 1:**
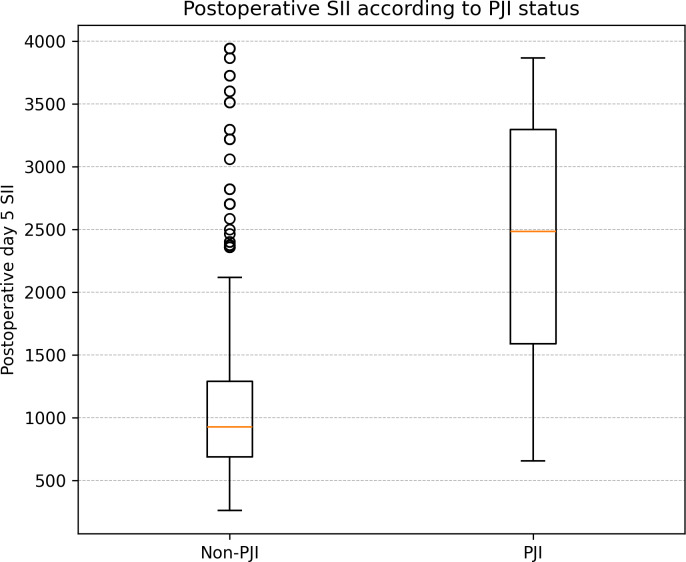
Postoperative SII according to PJI status. Values are presented as median and interquartile range, with whiskers indicating variability and circles representing outliers.

Baseline demographic and clinical characteristics of the study population stratified by PJI status are presented in Table 2. Patients who developed PJI had significantly higher postoperative day-5 SII values compared to those who did not develop infection (Fig. 1). In addition, body mass index and the prevalence of diabetes mellitus were significantly higher in the PJI group. No statistically significant differences were observed between groups in terms of sex, Charlson comorbidity index, or fracture characteristics.

**Table 2:** Baseline characteristics according to PJI status.

Variable	PJI ( n=47 )	Non-PJI ( n=929 )	p value
Age (years), mean ± SD	82.1±6.8	80.3±6.2	0.086
Male sex, n (%)	12 (25.5)	313 (33.7)	0.318
BMI, mean ± SD	38.1±4.0	28.6±5.9	<0.001
ASA score, n (%)			0.611
ASA 2	0 (0.0)	4 (0.4)	
ASA 3	46 (97.9)	917 (98.7)	
ASA 4	1 (2.1)	8 (0.9)	
Charlson comorbidity index, mean ± SD	4.7±2.4	4.3±3.5	0.275
Diabetes mellitus, n (%)	22 (46.8)	266 (28.6)	0.012
Length of hospital stay (days), median (IQR)	5.0 (5.0–6.0)	5.0 (5.0–6.0)	0.694
Preoperative SII, mean ± SD	1308.0±860.7	1164.7±859.1	0.270
Postoperative day-5 SII, mean ± SD	2465.2±984.1	1070.8±601.9	<0.001

In univariable logistic regression analysis, postoperative SII, body mass index, and diabetes mellitus were significantly associated with the development of PJI. Preoperative SII and length of hospital stay were not significantly associated with PJI risk (Table 3, part A).

**Table 3:** Logistic regression analyses.

Variable	OR (95 % CI)	p value
A. Univariable analysis		
Postoperative SII (per 100 units)	1.174 (1.138–1.212)	<0.001
Preoperative SII (per 100 units)	1.018 (0.987–1.050)	0.266
Δ SII (per 100 units)	1.121 (1.089–1.154)	<0.001
Age (years)	1.047 (0.998–1.097)	0.060
BMI	1.274 (1.205–1.348)	<0.001
ASA score	2.706 (0.381–19.232)	0.320
Charlson comorbidity index	1.022 (0.966–1.082)	0.450
Diabetes mellitus	2.193 (1.215–3.958)	0.009
Length of hospital stay (days)	1.077 (0.751–1.544)	0.689
Male sex	0.675 (0.345–1.318)	0.250
B. Multivariable analysis (primary model)		
Postoperative SII (per 100 units)	1.195 (1.144–1.248)	<0.001
Age (years)	1.057 (0.993–1.125)	0.083
BMI	1.297 (1.204–1.397)	<0.001
ASA score	0.073 (0.005–0.982)	0.048
Charlson comorbidity index	1.016 (0.938–1.100)	0.693
Diabetes mellitus	3.020 (1.326–6.876)	0.008
Length of hospital stay (days)	1.109 (0.683–1.800)	0.676
Male sex	0.851 (0.349–2.073)	0.722
C. Multivariable analysis ( Δ SII model)		
Δ SII (per 100 units)	1.124 (1.085–1.164)	<0.001
Age (years)	1.070 (1.011–1.132)	0.019
BMI	1.286 (1.205–1.373)	<0.001
ASA score	0.239 (0.017–3.403)	0.291
Charlson Comorbidity Index	1.010 (0.932–1.095)	0.806
Diabetes mellitus	2.247 (1.069–4.724)	0.033
Length of hospital stay (days)	0.963 (0.611–1.519)	0.872
Male sex	0.754 (0.335–1.694)	0.494

Multivariable logistic regression analysis was performed to evaluate independent predictors of PJI, adjusting for clinically relevant confounders including age, sex, body mass index, ASA score, Charlson comorbidity index, diabetes mellitus, and length of hospital stay (Table 3, part B). Postoperative SII remained an independent predictor of PJI, with higher values associated with increased risk of infection. Body mass index and diabetes mellitus also demonstrated independent associations with PJI, while other variables did not retain statistical significance in the adjusted model.

**Figure 2:**
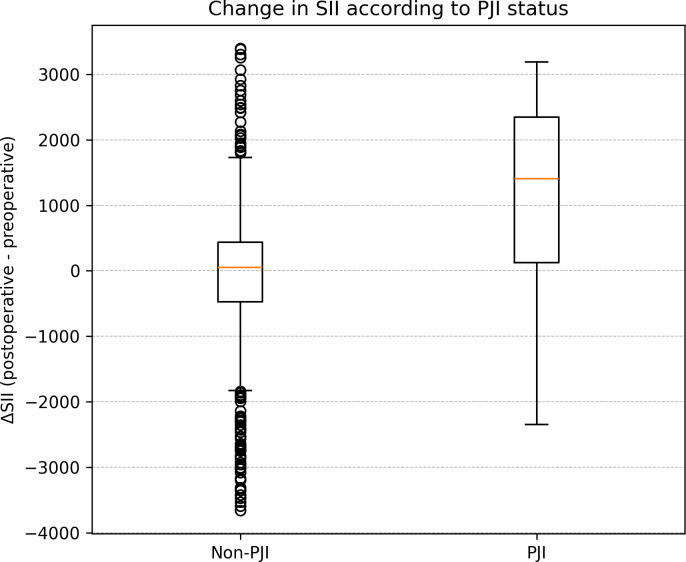
Change in SII ( Δ SII) according to PJI status. Values are presented as median and interquartile range, with whiskers indicating variability and circles representing outliers.

To further evaluate the dynamic inflammatory response, the change in SII between preoperative and postoperative measurements ( Δ SII) was analysed. Δ SII was significantly associated with PJI in both univariable and multivariable models, suggesting that postoperative elevation relative to the baseline provides additional prognostic information (Fig. 2, Table 3, part C).

**Figure 3:**
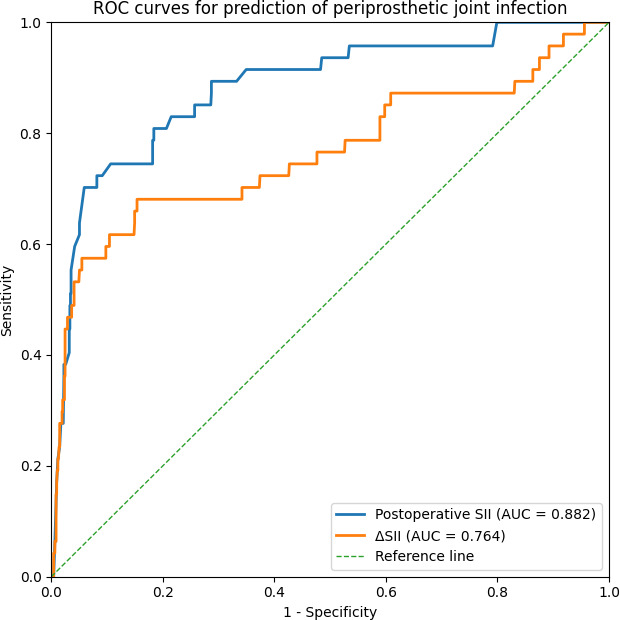
ROC curves of postoperative SII and Δ SII.

ROC curve analysis demonstrated good discriminatory ability of postoperative SII for predicting PJI, with an area under the curve (AUC) of 0.882 (95 % CI 0.823–0.932). The optimal cut-off value, determined using the Youden index, was 2118, yielding a sensitivity of 70.2 % and a specificity of 94.1 % (Fig. 3, Table 4).

**Table 4:** Diagnostic performance of postoperative SII.

Parameter	Value
AUC	0.882
95 % CI	0.823–0.932
Optimal cut-off (Youden index)	2118
Sensitivity	70.2 %
Specificity	94.1 %
Positive predictive value (PPV)	37.5 %
Negative predictive value (NPV)	98.4 %

ROC analysis was also performed for Δ SII. The AUC was 0.764 (95 % CI 0.667–0.852), indicating moderate discriminatory performance for predicting PJI. The optimal cut-off value determined by the Youden index was 656, yielding a sensitivity of 68.1 %, specificity of 84.6 %, positive predictive value (PPV) of 18.3 %, and negative predictive value (NPV) of 98.1 % (Fig. 3, Table 5).

**Table 5:** Diagnostic performance of Δ SII.

Parameter	Value
AUC	0.764
95 % CI	0.667–0.852
Optimal cut-off (Youden index)	656
Sensitivity	68.1 %
Specificity	84.6 %
Positive predictive value (PPV)	18.3 %
Negative predictive value (NPV)	98.1 %

## 4 Discussion

In this study, we found that postoperative SII is significantly associated with the development of PJI and remains an independent predictor after adjustment for relevant clinical confounders. In addition, Δ SII was also associated with PJI, suggesting that changes in inflammatory status between the preoperative and postoperative periods may be relevant when evaluating infection risk.

When demographic and clinical characteristics were examined, body mass index and diabetes mellitus were found to be significantly associated with PJI, whereas age, sex, Charlson comorbidity index, and length of hospital stay were not. The association between obesity and increased risk of postoperative infection has been consistently reported in the literature, likely related to impaired wound healing, altered immune response, and increased surgical complexity (Chang and Peng, 2023). The relatively higher BMI observed in the PJI group should be interpreted with caution as it may reflect underlying selection bias inherent to retrospective analyses. Similarly, diabetes mellitus is a well-established risk factor for infection due to its effects on microvascular circulation and immune dysfunction (Casqueiro et al., 2012).

In contrast, age, sex, Charlson comorbidity index, and length of hospital stay were not significantly associated with PJI in our cohort. However, findings regarding other demographic factors remain inconsistent across studies. In a recent study, male sex, diabetes mellitus, and younger age were associated with an increased risk of prosthetic joint infection following total knee arthroplasty (Chou et al., 2026). While our findings are in line with the association observed for diabetes mellitus, the association between age and PJI was not definitive. Older patients appeared to have a higher risk of infection, although statistical significance was not reached. We did not observe a significant relationship between sex and PJI. These discrepancies likely reflect differences in patient populations, surgical procedures, and baseline risk profiles. In contrast, the lack of association between age and PJI in our cohort is consistent with several studies focusing on geriatric populations, where age alone may be less discriminatory once a certain threshold is reached (Inoue et al., 2019). The absence of a significant relationship between length of hospital stay and PJI further suggests that prolonged hospitalization may reflect the consequences rather than the primary drivers of postoperative complications (Lenguerrand et al., 2018).

The relatively short hospital stay observed in our cohort may be related to the organization of care at our institution. As a high-volume trauma centre, patients are managed through a well-established postoperative pathway, and discharge planning is typically initiated early after surgery. Rehabilitation needs are also addressed during the early postoperative period whenever possible. As a result, length of stay tends to be relatively consistent across patients and may reflect local clinical practice rather than differences in individual risk profiles.

In univariable analysis, postoperative SII, BMI, and diabetes mellitus were significantly associated with PJI. Notably, postoperative SII was associated with infection risk, whereas preoperative SII was not, suggesting that inflammatory changes occurring after surgery may be more informative than preoperative measurements alone.

Multivariable analysis further strengthened these findings, demonstrating that postoperative SII remained an independent predictor of PJI after adjustment for clinically relevant confounders. The persistence of this association after adjustment suggests that postoperative SII captures information that is not fully explained by conventional clinical risk factors. Previous studies have also reported that SII may reflect clinically relevant inflammatory changes across different patient populations (Zhu et al., 2026; Moldovan, 2024). Because it incorporates changes in neutrophil, lymphocyte and platelet counts into a single measure, SII may provide a broader reflection of the host response following surgery. This may partly explain why higher postoperative SII values were observed among patients who subsequently developed infection.

The analysis of Δ SII provided additional insight into the dynamic nature of the inflammatory response. The independent association observed for Δ SII supports the concept that perioperative changes, rather than static measurements, may better reflect the pathophysiological processes associated with increased infection risk. An exaggerated increase in inflammatory markers following surgery may indicate a dysregulated host response to surgical stress, which could predispose patients to infectious complications (Desborough, 2000; Gollwitzer et al., 2013). Evidence regarding Δ SII in orthopaedic patients remains scarce. However, comparable observations have been reported in studies examining postoperative inflammatory changes in other surgical populations (Chung et al., 2025). Although Δ SII was significantly associated with PJI, postoperative SII demonstrated greater discriminatory ability. This may indicate that the inflammatory status measured after surgery carries more clinically relevant information than the degree of change from the baseline alone.

Most infections in the present study occurred within the early postoperative period. *Staphylococcus aureus* and *Staphylococcus epidermidis* were the predominant microorganisms identified among infected patients. This pattern suggests that the observed inflammatory response was mainly associated with pathogens that are frequently encountered in routine arthroplasty practice (van Veghel et al., 2025).

ROC analysis demonstrated that postoperative SII was able to differentiate patients who developed PJI from those who remained infection-free with good accuracy. The high negative predictive value observed in our cohort suggests that patients with lower postoperative SII values were unlikely to develop infection during follow-up. Although the identified cut-off may help in the interpretation of postoperative inflammatory findings, SII should be evaluated together with clinical assessment and other available investigations rather than being considered a standalone indicator of infection (Shahi and Parvizi, 2017).

Several limitations of this study should be considered. First, the retrospective single-centre design limits causal inference and may affect generalizability. Although multiple clinically relevant variables were included, residual confounding cannot be excluded, particularly from unmeasured factors such as nutritional status, perioperative management, and functional capacity.

Another limitation is that commonly used inflammatory markers, including C-reactive protein and erythrocyte sedimentation rate, were not routinely measured unless clinically indicated. As a result, we were unable to directly compare SII with these conventional biomarkers.

The distribution of ASA scores was highly unbalanced, with most patients being classified as ASA 3. Consequently, regression estimates related to ASA should be interpreted with caution as the limited number of patients in the ASA 2 and ASA 4 categories may have affected coefficient stability.

Similarly, the relatively narrow range of hospital stay durations in our cohort may have limited our ability to detect a potential association between length of stay and PJI.

Postoperative clinical decisions were not guided by SII values during the study period. While this may be considered to be a limitation, it also reduces the likelihood of treatment-related bias and reflects the natural relationship between postoperative inflammatory response and infection development.

In addition, some variables, most notably transfusion status, showed inconsistencies during data validation and were excluded from the primary analysis.

Despite these limitations, the study has several strengths. The relatively large sample size and homogeneous geriatric population strengthen internal consistency. The evaluation of both static and dynamic SII parameters provides a broader assessment of the inflammatory response, and the use of multivariable models allows a more reliable evaluation of independent associations.

## 5 Conclusions

In conclusion, our findings suggest that postoperative systemic inflammatory burden, as reflected by SII, is associated with the development of periprosthetic joint infection following hemiarthroplasty in elderly patients. Notably, the change in SII from the preoperative to postoperative period appears to provide additional insight beyond single measurements, supporting the idea that dynamic inflammatory responses may better capture infection risk. Given that SII is derived from routinely available blood parameters, it may offer a practical adjunct for early postoperative risk assessment. However, these findings should be interpreted in the context of overall clinical evaluation, and further prospective studies are needed to clarify its role in guiding clinical decision-making.

## Data Availability

The datasets generated and analysed during the current study are not publicly available because they contain patient-related information and are subject to institutional and ethical restrictions. Data are available from the corresponding author on reasonable request and with permission from the relevant institutional authorities.

## Code availability

No custom code was used in this study.
